# (−)-Epicatechin Reduces Neuroinflammation, Protects Mitochondria Function, and Prevents Cognitive Impairment in Sepsis-Associated Encephalopathy

**DOI:** 10.1155/2022/2657713

**Published:** 2022-05-24

**Authors:** Jianmin Ling, Yanqing Wu, Xiaojing Zou, Yanmin Chang, Gang Li, Minghao Fang

**Affiliations:** ^1^Emergency Department and Intensive Care Unit, Tongji Hospital, Tongji Medical College, Huazhong University of Science and Technology, Wuhan 430030, China; ^2^Department of Neurology, Union Hospital, Tongji Medical College, Huazhong University of Science and Technology, Wuhan 430030, China

## Abstract

Sepsis-associated encephalopathy is a common neurological complication of sepsis. Despite advances in pathological and diagnostic investigations, its treatment remains a major challenge. In sepsis-associated encephalopathy, neuroinflammatory overactivation and mitochondrial damage are thought to contribute to cognitive and behavioral impairments. In this study, we found that administration of (−)-Epicatechin, a dietary flavonoid of the flavan-3-ol subgroup, improves memory deficits and behavior performance by ameliorating neuroinflammation, regulating mitochondria function, enhancing synaptic plasticity, and reducing neuronal loss in a mouse model of lipopolysaccharide-induced sepsis. We further show that the AMPK signaling pathway might be among the mechanisms involved in the beneficial memory effects. Our data demonstrated the potential of (−)-Epicatechin as a new drug candidate for the treatment of sepsis-associated cognitive impairment by targeting AMPK.

## 1. Introduction

Sepsis is a common condition associated with severe multiorgan failure and high mortality [1]. Short- or long-term neurological dysfunction is frequently observed in patients with sepsis [2]. Sepsis-associated encephalopathy (SAE) refers to acute diffuse brain dysfunction that arises in the context of sepsis without an overt central nervous system (CNS) infection [3]. SAE is common in an intensive care unit (ICU), and up to 70% of sepsis-associated nonsurvivors experience severe SAE [4]. SAE-associated cognitive deficits contribute to the decrease of the ability of people to live independently [5]. There is no definitive therapy for SAE; therefore, new therapies are required.

(−)-Epicatechin (Epi), which belongs to the polyphenol flavonoid group, is reported to improve cognitive impairment and behavioral performance through the regulation of biomarkers involved in neuroinflammation and restoration of mitochondrial defects in mice [6–8]. Epi also reportedly has various biological activities that mainly depend on the stimulation of critical factors in the adenosine 5′-monophosphate-activated protein kinase (AMPK), nuclear factor kappa-B (NF-*κ*B), nitric oxide (NO), and phosphatidylinositol 3 kinase (PI3K)/protein kinase B (AKT) pathways [9]. AMPK regulates energy metabolism, and activation of AMPK increases the rate of ATP-generating. NF-*κ*B is a mediator that is of central importance in the proinflammatory response. NO plays an important role in the immune system. The PI3K/AKT pathway regulates the signal transduction, cell proliferation, and apoptosis process. Several preclinical and clinical studies have been conducted on the role of Epi in neurodegeneration, cardiovascular, and cerebrovascular diseases, such as Alzheimer's disease, stroke, and atherosclerosis [10–13]. Moreover, Epi mitigates the inflammatory response in lipopolysaccharide- (LPS-) induced mouse models of renal and lung injury [14, 15].

Some researches proved that AMPK played a protective role in sepsis via activating the downstream effector molecules such as sirt1, sirt3, and Parkin [16–18]. Loss of AMPK activity has been reported in sepsis, which may contribute to disturbing mitochondrial function and exacerbate neuroinflammation [19, 20]. Activation of AMPK promotes mitochondrial biogenesis and alleviates sepsis [16]. Targeted activation of AMPK ameliorated sepsis-induced inflammation and apoptosis [21]. Upregulating AMPK exerts anti-inflammatory and antioxidant effects in sepsis-mediated brain injury by repressing the MAPK and NF-*κ*B pathways [22].

Owing to its antioxidant and anti-inflammation effects, Epi has great potential for use in sepsis. However, to date, no study has investigated the effects of Epi on neuroinflammation, mitochondrial damage, or behavioral deficits associated with SAE. Therefore, this study is aimed at ascertaining whether Epi might affect spatial memory induced by sepsis. In humans, sepsis is a heterogeneous disease. It is often modeled in mice via the intraperitoneal injection of the bacterial endotoxin LPS. To this end, we explored the effects of Epi administration in an LPS-induced model of sepsis [23, 24] and measured hippocampal-dependent spatial memory, microgliosis, inflammatory mediators, mitochondrial damage, and possible mechanisms.

## 2. Materials and Methods

### 2.1. Compounds and Antibodies

The compounds and antibodies used are as follows: (−) − Epicatechin ≥ 90% (HPLC, EC, Sigma, USA, E1753), lipopolysaccharide (LPS, Sigma, USA, L2654), Compound C (dorsomorphin, APExBIO, USA, B1372), antibodies including rabbit polyclonal anti-IL-6 antibody (ProteinTech, China, 21865-1-AP, 1 : 500), rabbit monoclonal anti-IL-1*β* antibody (Abcam, USA, ab200478, 1 : 500), mouse monoclonal anti-TNF*α* antibody (ProteinTech, China, 60291-1-Ig, 1 : 500), rabbit monoclonal anti-AMPK antibody (Abcam, USA, ab32047, 1 : 1000), rabbit monoclonal anti-pAMPK antibody (Cell Signaling Technology, USA, 2535S, 1 : 1000), rabbit monoclonal antisynaptophysin antibody (Abcam, USA, ab32127, 1 : 1000), rabbit polyclonal anti-PSD95 antibody (Abcam, USA, ab18258, 1 : 1000), rabbit monoclonal anti-TOMM20 antibody (Abcam, USA, ab186735, 1 : 500), rabbit polyclonal anti-Iba1 antibody (Wako, Japan, 019-19741, 1 : 100), mouse monoclonal anti-GAPDH antibody (ProteinTech, China, 60004-1-AP, 1 : 5000), and mouse monoclonal anti-DM1A antibody (Abcam, USA, ab7291, 1 : 1000).

### 2.2. Cell Lines and Culture Conditions

BV2 microglial cells (a generous gift from Dr. Bingge Zhang, Huazhong University of Science and Technology) were maintained in high-glucose DMEM (Thermo Fisher, D2650) with 5% fetal bovine serum (FBS, Thermo Fisher, 16140071) in a humidified incubator at 37°C under 5% CO_2_ containing atmosphere.

### 2.3. Animals and Drug Administration

Wild-type C57BL/6 mice (8 wk old, *n* = 45, male) were purchased from *Beijing Vital River Laboratory*. Sepsis was induced by the LPS (5 mg/kg) model [24]. LPS mice were further randomized to receive saline (0.1 mL/mouse, gavage) or Epi (50 mg/kg, gavage) after 0.5, 24, and 48 hours. Twenty-four hours after LPS induction, the murine sepsis score (MSS) was calculated based on the parameters in the previous researches described [2, 25, 26]. Evaluators were blinded to group assignments. All mice were kept at a specific pathogen-free environment feeding center, with a 12 h light/dark cycle, an appropriate temperature, and plenty of food and water. The animal experiments conducted were approved by the Institutional Animal Care and Use Committee at Tongji Medical College, Huazhong University of Science and Technology.

### 2.4. Cells Viability Assay

The viability of BV2 cells to Epi exposure was measured by using the CCK-8 assay kit (Dojindo, Kumamoto, Japan). The BV2 cells (5 × 10^3^ cells/well) were plated onto a 96-well plate overnight. Then, the cells were treated with a final concentration of 0, 50, 100, 200, 500, or 1000 *μ*M/mL for 24 h. The CCK-8 assay buffer was prepared by following the manufacturer's instructions [27]. Then the CCK-8 test solution was added to each well and was incubated for 2 h at 37°C. The absorbance at 450 nm was detected using a microplate reader (Bio-Rad, Hercules, USA). The experiment was repeated three times.

### 2.5. Western Blotting

Biological extractions of BV2 cells and hippocampus were performed using RIPA lysis buffer, 1 mM PMSF, and 1× protease inhibitor cocktail (Sigma) as described previously [28]. The concentration of protein in the samples was determined using a Pierce™ BCA protein assay kit (Thermo Scientific™, USA). In brief, a total of 10-30 *μ*g protein extracted was separated by 10-12% SDS-PAGE and transferred to nitrocellulose blotting membranes (Amersham Biosciences, Germany), blocked with 5% BSA for 1 h, and followed by incubating with primary antibodies overnight at 4°C. After that, the membranes were incubated with IRDyeTM- (800CW) conjugated anti-mouse or anti-rabbit secondary antibody for 1.5 h at room temperature. Signals were detected with the Bio-Rad imaging system (USA) or Odyssey System (USA). The densitometry was analyzed by ImageJ (USA).

### 2.6. qRT-PCR Analysis

Total RNA was extracted from hippocampus tissues using a TaKaRa (Dalian, China) RNAiso Reagent (TRIzol) [29]. cDNA synthesis was performed using a PrimeScript™ RT reagent kit (Takara) according to the manufacturer's instructions. All PCR reactions used SYBR Green Master Mix (Takara, Japan), primers (Tsingke Biotechnology, Beijing, China), and cDNA to a final volume of 10 *μ*L, with each sample performed in triplicate in the StepOnePlus Real-Time PCR System (Thermo Fisher, USA) according to the protocol of the manufacturer. The following additional primers were used: IL-6 forward 5′-AGTGGCTAAGGACCAAGAC-3′, reverse 5′-ATAACGCACTAGGTTTGCCGA-3′; IL-1*β* forward 5′-GCACTACAGGCTCCGAGATGAA-3′, reverse 5′-GTCGTTGCTTGGTTCTCCTTGT-3′; TNF*α* forward 5′-CACGCTCTTCTGTCTACTGAACTTC-3′, reverse 5′-ATGATCTGAGTGTGAGGGTCTGG-3′; and *β*-actin forward 5′-GGCTGTATTCCCCTCCATCG-3′, reverse 5′-CCAGTTGGTAACAATGCCATGT-3′. Total cell and mice RNA levels were normalized to mouse *β*-actin mRNA and quantified by the 2^-*ΔΔ*Ct^ method for relative expression analysis. Abdominal brain mt DNA-CN was estimated by qRT-PCR, and relative amounts of ND1/18S or ND4/18S were compared as previously described [30]. The following additional primers were used: mt ND1 forward 5′-CAGCCGGCCCATTCGCGT TA-3′, reverse 5′-AGCGGAAGCGTGGATAGG ATGC-3′; mt ND4 forward 5′-TCGCCTACTCCTCAGTTA GCC ACA-3′, reverse 5′-TGATGATGTGAGGCCATGTGCGA-3′; and 18S forward 5′-GGGAGCCTGAGAAACGGC-3′, reverse 5′-GGGTCGGGAGTGGGTAATTT-3′.

### 2.7. Measurement of Adenosine Triphosphate (ATP) Levels

The ATP levels of BV2 cells and hippocampus tissues were measured using an ATP test kit by following the previously described protocol (S0026, Beyotime, China) [31]. The resulting luminescence was measured at 562 nm with Synergy2 (BioTek, USA). ATP levels were calculated in nmol/mg.

### 2.8. Measurement of Malondialdehyde (MDA) Productions

The Lipid Peroxidation Assay Kit (S0131S, Beyotime, China) was used to measure MDA in BV2 cells and hippocampus tissues [32]. Briefly, cells or tissues were extracted as previously described, and then samples (100 *μ*L) were mixed with MDA detection fluid (200 *μ*L) in a boiling water bath for 15 min. Finally, each sample and standard (200 *μ*L) were added to a 96-well plate, and the absorbance was measured at 532 nm by a microplate reader.

### 2.9. Measurement of SOD Activity

The activities of SOD in the hippocampus were measured using a SOD kit (S0101M, Beyotime, China) according to the manufacturer's instructions [32]. Add the SOD sample preparation solution provided by this kit according to the protocol for perfusion of brain samples. Use the BCA method to measure the sample concentration before adding the WST-8/enzyme working solution. One unit of SOD activity was defined as the amount that reduced the absorbance at 450 nm by 50%.

### 2.10. Mitochondrial Membrane Potential (MMP)

A JC-1 assay kit (C2003S, Beyotime, China) was used to measure MMP in vitro [33]. In brief, cells were treated with JC-1 staining working solution for 20 minutes in a humidified incubator at 37°C under a 5% CO_2_-containing atmosphere and analyzed by fluorescence confocal microscopy (LSM 800, Zeiss, Germany).

### 2.11. Behavioral Tests

#### 2.11.1. Novel Object Recognition (NOR)

The tests were carried out in an open field arena measuring 50 (*w*) × 50 (*d*) × 50 (*h*) cm [34]. The mice were acclimated to the recognition room 24 h prior to testing. On the training day, mice were placed into the center of the area in the presence of two identical objects. Every mouse was allowed to freely explore for 10 minutes. The interaction which was defined as nasal or oral contact with an object was recorded by a video tracker (Chengdu Taimeng Software Co., Ltd, China). 24 hours after training, mice were reinserted into the arena for free exploration for 10 min again, in which one of the objects had already been replaced by a novel object. Results were expressed as a preferential index (PI): PI = novel object exploration time/(total exploration time) × 100%.

#### 2.11.2. Morris Water Maze (MWM)

To detect the spatial memory and learning ability of mice, the MWM test was performed as previously reported [35]. The mice were trained to locate a platform hidden 1.5 cm underwater in the maze with a diameter of 120 cm and a high of 60 cm. The maze was divided into 4 quadrants. Mice were trained to find the platform for 5 consecutive days, 3 trials per day with a 20 s interval from 8:00 to 12:00 a.m. The swimming tracks were video recorded in each training session by a digital device. On the 7^th^ day, the platform was removed from the pool, and the probe test was performed. During the test, escape latency, time in the target quadrant, and mean annual crossings were video tracked using the Chengdu Taimeng Software Co. Ltd (China).

### 2.12. Nissl Staining

The brain tissue of mice used for Nissl staining was slid with 5 *μ*m sections and stained with Nissl staining solution (Servicebio, GP1043) for 2-5 min and then washed by running water until colorless. Use 0.1% glacial acetic acid to differentiate the sections until the Nissl body was dark blue, and the background was light blue or colorless. The slides were dried and coverslipped with Permount TM Mounting Medium [36].

### 2.13. Golgi Staining

Dendritic spines were studied in the hippocampi of mice by FD Rapid GolgiStain™ kit (USA) [37]. In brief, brain tissues were soaked in equal volumes of Solutions A and B which were mixed one day in advance and then were incubated away from light at room temperature for 3 weeks. The brain tissues were then transferred to Solution C twice for 1 week. Next, the tissues were sliced into 100 *μ*m-thick sections. Then, the sections were rinsed with Milli-Q water 2 times, 4 min each, and incubated in a mixture of Solutions D and E for 10 min. The sections were dehydrated by ethanol in series, cleared in xylene, covered slips with Permount, dried at room temperature, and observed under the Nikon microscope (Tokyo, Japan).

### 2.14. Transmission Electron Microscopy (TEM)

For quantitative analysis of the mitochondria by electronic microscopy, the mice were perfused with PBS, followed by 2.5% glutaraldehyde-4% paraformaldehyde in phosphate buffer (pH 7.4). The brains were then removed and were postfixed in the same fixative overnight at 4°C. After that, tissues were cut into thick slices (50 mm). The hippocampi were processed by postfixation in 1% osmium tetroxide for 25 min, dehydrated with alcohol, and embedded in epoxy resin. The selected areas were cut into ultrathin sections (60 nm). Ultrathin sections were stained with uranyl acetate and lead citrate before imaging with the JEM-1400 electron microscope (Japan).

### 2.15. Immunofluorescence Staining on Mouse Samples

Mice were anesthetized and transcardially perfused with 4% paraformaldehyde. In order to prepare 5 *μ*m-thick coronal sections of the hippocampus, the brains were taken to paraffin embedding and cut into 5 *μ*m-thick slices on a microtome (Leica RM 2245). Before staining, the slices underwent dewaxing, hydration, and a series of antigen retrieval. The sections were preincubated in 0.3% hydrogen peroxide for 30 min and then moved on to 1× PBS, 0.5% Triton X-100, and 5% BSA. Next, hippocampus sections were incubated with primary antibodies on a shaker at 4°C overnight. Appropriate secondary antibodies were incubated for 1 h at 37°C.

Slides were sealed with nail polish and stored at 4°C. The images were captured by Nikon SV120 (Tokyo, Japan).

### 2.16. Statistical Analysis

Data were analyzed using GraphPad Prism 8. The results were presented as mean ± standard error of the mean (SEM). Comparisons between groups were performed using a *t*-test, one-way ANOVA test, or two-way repeated-measures ANOVA test followed by Tukey's post hoc test. *P* values less than 0.05 indicated statistical significance.

## 3. Results

### 3.1. Epi Suppresses LPS-Induced Inflammatory Responses in the Culture of BV2 Cells

**Figure 1:**
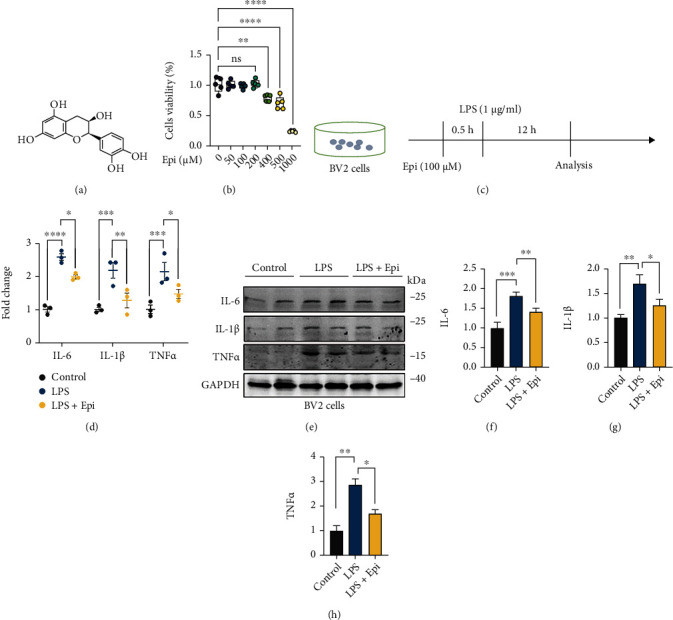
Effects of Epi on LPS-induced inflammatory cytokine production. (a) Chemical structure of (−)-Epicatechin. (b) Effects of different concentrations of Epi alone on cell viability. (c) Experimental scheme: BV2 microglial cells were pretreated with DMSO or Epi for 0.5 h, and then cells were treated with normal saline or LPS for 12 h. (d) Expression of IL-6, IL-1*β*, and TNF*α* at the mRNA level in cells 12 h after LPS induction (*n* = 3 for each group). (e–h) Determination of IL-6, IL-1*β*, and TNF*α* protein levels by western blot analysis. *n* = 3 for each group. All data are shown as mean ± SEM. One-way ANOVA test followed by Tukey's post hoc test, ^∗^*p* < 0.05, ^∗∗^*p* < 0.01, ^∗∗∗^*p* < 0.001, ^∗∗∗∗^*p* < 0.0001.

In order to explore the role of Epi in the culture of a microglial cell line, BV2 cells were incubated with various doses of Epi, and the CCK8 assay was used to observe cell viability (Figure 1(a)). We found that Epi at various concentrations (0, 50, 100, and 200 *μ*M) had no significant effect on cell viability (Figure 1(b)); therefore, we chose an Epi concentration of 100 *μ*M for the subsequent Epi experiments in BV2 microglial cells. To explore the potential modifying functions of Epi on proinflammatory responses, BV2 microglial cells were treated according to the protocol described in Figure 1(c). Total RNA was extracted from samples, and proinflammatory cytokine levels were checked by qRT-PCR. The results showed that, compared with the control group, LPS increased the generation of proinflammatory cytokines (IL-6, IL-1*β*, and TNF*α*), while Epi induced a prominent reduction (Figure 1(d)). To further demonstrate the effects of Epi on LPS-induced inflammatory responses in the culture of BV2 cells, the levels of IL-6, IL-1*β*, and TNF*α* were confirmed by using western blot analysis (Figure 1(e)). The results showed that, compared with the control group, LPS increased the expressions of IL-6, IL-1*β*, and TNF*α*. Administration of Epi significantly decreased the levels of IL-6, IL-1*β*, and TNF*α* compared with the LPS group (Figures 1(e)–1(h)), consistent with the qRT-PCR results. The above results demonstrate that the administration of Epi attenuated the generation of proinflammatory cytokines in LPS-challenged microglia.

### 3.2. Epi Decreases Mitochondria Damage

**Figure 2:**
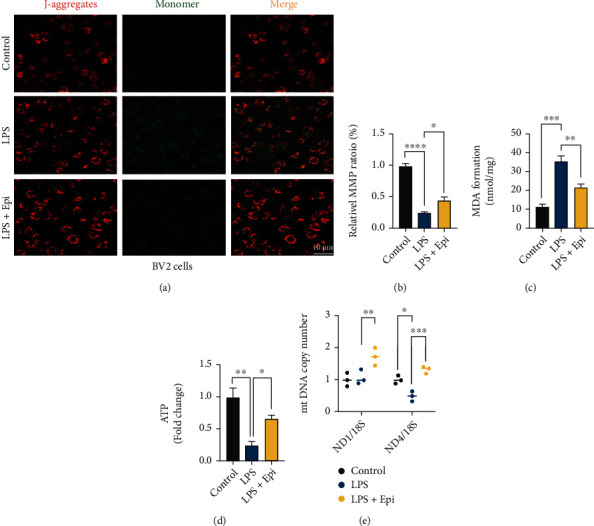
Epi protected LPS-induced mitochondrial dysfunction in vivo. (a, b) The MMP was evaluated using JC-1 staining followed by fluorescence microscopy. JC-1 exists as a monomer and was shown in red in the mitochondrial matrix when the MMP is high, whereas it exists in the aggregate form out of mitochondria and was shown in green when the MMP is low. (c, d) ATP and MDA levels were detected. (e) Epi increased mt DNA copy number in LPS-induced cells by using qRT-PCR analysis. *n* = 3 for each group. All data are shown as mean ± SEM. One-way ANOVA test followed by Tukey's post hoc test, ^∗^*p* < 0.05, ^∗∗^*p* < 0.01, ^∗∗∗^*p* < 0.001, ^∗∗∗∗^*p* < 0.0001.

Mitochondrial dysfunction may drive inflammation activation [38]. To verify the effects of Epi on mitochondrial damage induced by LPS in BV2 microglia, we examined mitochondrial membrane potential (MMP) and the expression of malondialdehyde (MDA) and ATP using the protocol described in Figure 1(c). As shown in Figure 2(a), the fluorescence of BV2 cells in the LPS group changed to green relative to the control group, and the red/green fluorescence ratio decreased, which suggested that the MMP of BV2 cells declined after LPS injury and mitochondria were impaired. In comparison with the LPS group, the red/green fluorescence ratio was upregulated in the Epi group (Figures 2(a) and 2(b)). LPS-treated cells exhibited increased MDA generation and decreased ATP production, suggesting a decrease in antioxidant capacity and energy crisis following LPS treatment (Figures 2(c) and 2(d)). Administration of Epi ameliorated LPS-induced oxidative damage and mitochondrial dysfunction, as evidenced by the decreased MDA level and increased ATP production in Epi-treated BV2 cells. To investigate further, the mitochondrial copy number (mt DNA-CN) was measured by a qRT-PCR method following mitochondrial damage with LPS. After LPS treatment, ND4/18S ratio was degraded in BV2 cells, indicating reduced mitochondrial quantity. However, the ND1/18S ratio did not decrease following LPS treatment. In comparison with the LPS group, mt DNA-CN, including ND1/18S and ND4/18S, was higher in the Epi-treated group (Figure 2(e)). These data suggested that Epi improves LPS-induced mitochondrial dysfunction in BV2 cells.

### 3.3. Epi May Restore the LPS-Induced Mitochondria Dysfunction and Proinflammatory Responses by Activating AMPK In Vitro

**Figure 3:**
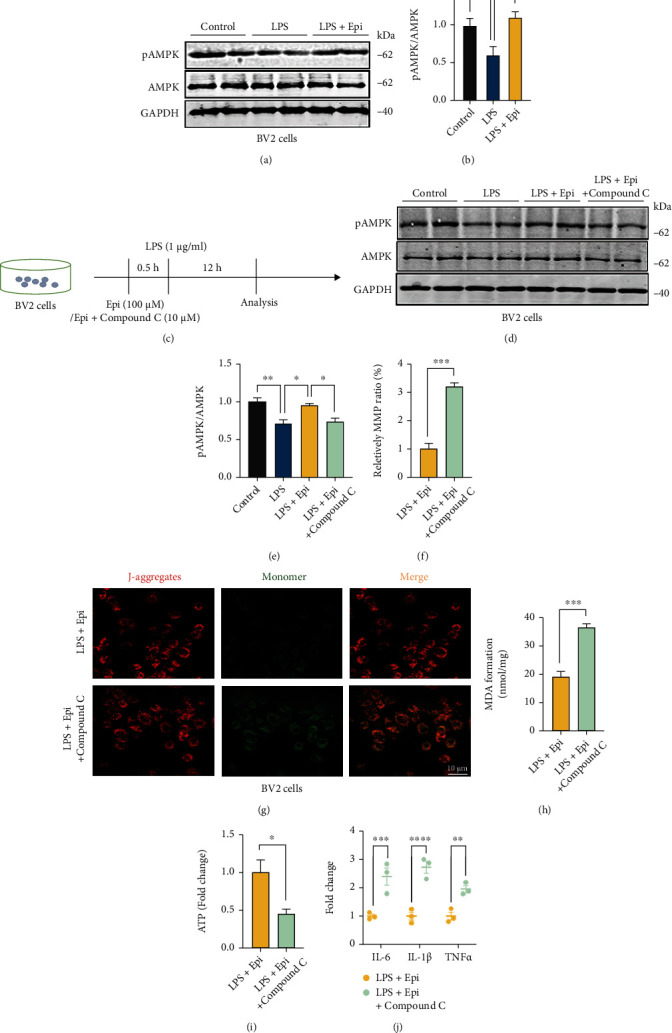
Epi modulates mitochondrial function and inflammatory response by activating AMPK. (a) Western blot analysis of the levels of pAMPK and AMPK in cells after treatment as described previously. Quantification of data is shown in (b). (c) Experimental scheme: BV2 microglial cells were pretreated with DMSO, Epi, or Epi with Compound C for 0.5 h, and then cells were treated with normal saline or LPS for 12 h. (d) Western blot analysis of the levels of pAMPK and AMPK in cells after treatment as described previously. Quantification of data is shown in (e). (f, g) The MMP was evaluated using JC-1 staining followed by fluorescence microscopy (g). Quantification of data is shown in (f). (h, i) ATP and MDA levels were detected. *n* = 3 for each group. (j) Expression of IL-6, IL-1*β*, and TNF*α* at the mRNA level in cells after administration of Epi or Epi with Compound C. *n* = 3 for each group. All data are shown as mean ± SEM. Unpaired student's *t*-test for (f), (h), and (i) and one-way ANOVA test followed by Tukey's post hoc test for the other analysis, ^∗^*p* < 0.05, ^∗∗^*p* < 0.01, ^∗∗∗^*p* < 0.001, ^∗∗∗∗^*p* < 0.0001.

AMPK deactivation plays a critical role in mitochondrial deficiency [39, 40]. To uncover the molecular causes of Epi-mediated restoration of LPS-induced mitochondrial impairment, we examined AMPK activity in BV2 cells. Western blot analysis showed a reduction in AMPK phosphorylation in response to LPS (Figure 3(a)). In contrast, we observed that phosphorylation levels were in the presence of Epi (Figures 3(a) and 3(b)). Next, we investigated whether AMPK inhibition could block the effect of Epi on LPS-induced mitochondrial dysfunction. In cells, the AMPK inhibitor Compound C was added to confirm the regulatory effect of Epi on mitochondria. The results showed that the inhibition of AMPK by cotreatment with LPS, Epi, and Compound C significantly decreased AMPK activity compared to that in LPS and Epi cotreated BV2 cells (Figures 3(c)–3(e)). In addition, Compound C treatment in LPS and Epi cotreated cells increased the levels of MMP and MDA and decreased the levels of ATP (Figures 3(f)–3(i)). To investigate the potential regulatory effects of Epi on proinflammatory responses by targeting AMPK, total RNA was isolated and proinflammatory cytokine levels were measured using qRT-PCR. The results showed that compared with the Epi-treated group, Epi+Compound C increased the generation of proinflammatory cytokines (IL-6, IL-1*β*, and TNF*α*) (Figure 3(j)). These results suggest that Epi may improve mitochondrial function and ameliorate inflammation in LPS-treated BV2 cells through the activation of AMPK.

### 3.4. Epi Rescues Memory Impairments In Vivo

**Figure 4:**
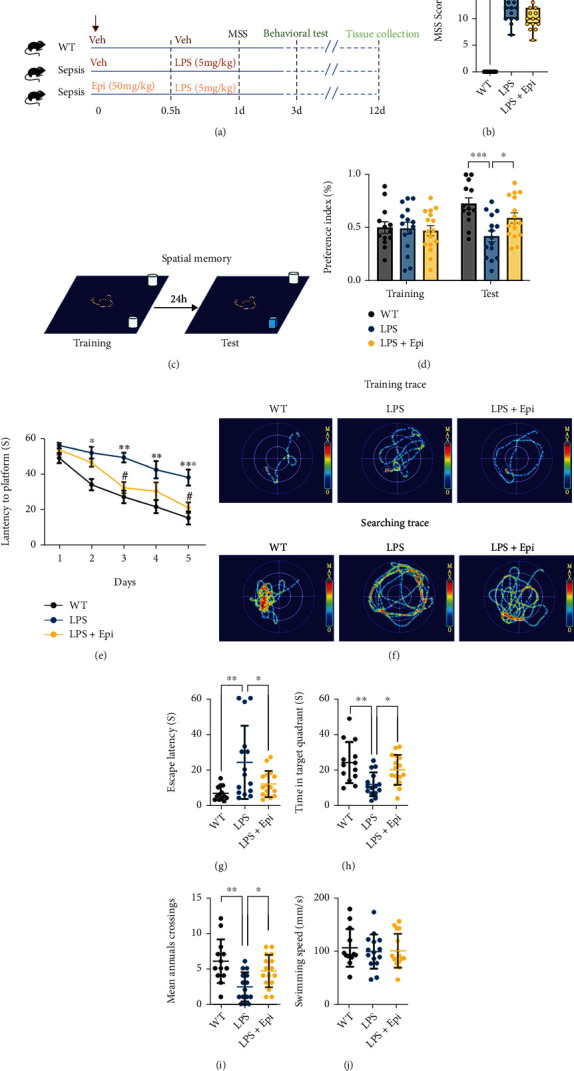
Epi attenuated cognitive impairments in LPS-induced mice. (a) Schematic illustration of the experimental design. Timeline for WT, LPS, or LPS+Epi mice. (b) LPS mice had a higher clinical score compared with the WT group at 24 hours. After therapy, the LPS+Epi group had a similar clinical score compared with LPS mice at 24 hours. (c) Pattern diagram for the new object recognition (NOR). (d) Preference index during training and test in NOR. (e) Latency to the platform during a 5-day training period of the Morris water maze (MWM). (f) The representative swimming trace of the mice during training and the test phase of MWM. (g) Escape latency, (h) time in the target quadrant, (i) times across the platform, and (j) swimming speed of mice. *n* = 13 − 16 mice/group. All data are shown as mean ± SEM. Two-way repeated-measures ANOVA test followed by Tukey's post hoc test for (e). One-way ANOVA test followed by Tukey's post hoc test for the other analysis, ^∗^*p* < 0.05, ^∗∗^*p* < 0.01, ^∗∗∗^*p* < 0.001, ^∗∗∗∗^*p* < 0.0001.

Neuroinflammation and mitochondrial dysfunction play important roles in the pathogenesis of SAE-associated cognitive impairment. After confirming that Epi could rescue LPS-induced inflammatory responses and mitochondrial damage in the culture of BV2 cells, we investigated whether Epi could improve LPS-induced cognitive deficits in vivo. The in vivo experiments were conducted in C57BL/6 mice following the administration of LPS and Epi (Figure 4(a)). C57BL/6J mice treated with normal saline were used as control. To identify the effects of Epi on LPS-induced mice, a clinical score analysis was conducted. LPS mice had a higher clinical score than the control group on day three (11.80 ± 0.6410 vs. 0); however, the LPS+Epi group did not have a lower clinical score after therapy (Figure 4(b)). Tests for cognitive function included a standard three-day novel object recognition (NOR) test and a seven-day Morris water maze (MWM). First, we tested hippocampal-dependent spatial memory based on the tendency of mice to explore novel objects, which reveals hippocampal integrity [41]. We first measured the exploration time for two identical objects during the first training day. The results showed that there was no significant difference in the time spent exploring two identical objects (Figures 4(c) and 4(d)). After 24 h, we found that the group of LPS mice showed a 1.7-fold reduction in exploration time of novel objects compared to that of vehicle- (normal saline) treated mice. However, we observed that Epi treatment increased the mean exploration time of novel objects by 1.3 times compared to that of LPS-treated mice in the NOR test (Figure 4(d)). During the MWM training, the LPS-treated mice showed learning and memory deficits as they took longer to find the hidden platform compared with the control group (Figures 3(e)–3(i)), while Epi improved learning ability both in the third and sixth days, shown by the decreased latency in time to find the platform (Figures 4(e) and 4(f)). In addition, the improved memory ability of Epi-treated mice was also shown by the decreased escape latency, the increased time spent in the platform quadrant, and more times crossing the platform area measured on the seventh-day test (Figures 4(f)–4(i)). However, no significant differences in the swimming speed were observed (Figure 4(j)).

### 3.5. Epi Ameliorates Neuronal Loss and Synaptic Disorder in LPS-Induced Mouse Model

**Figure 5:**
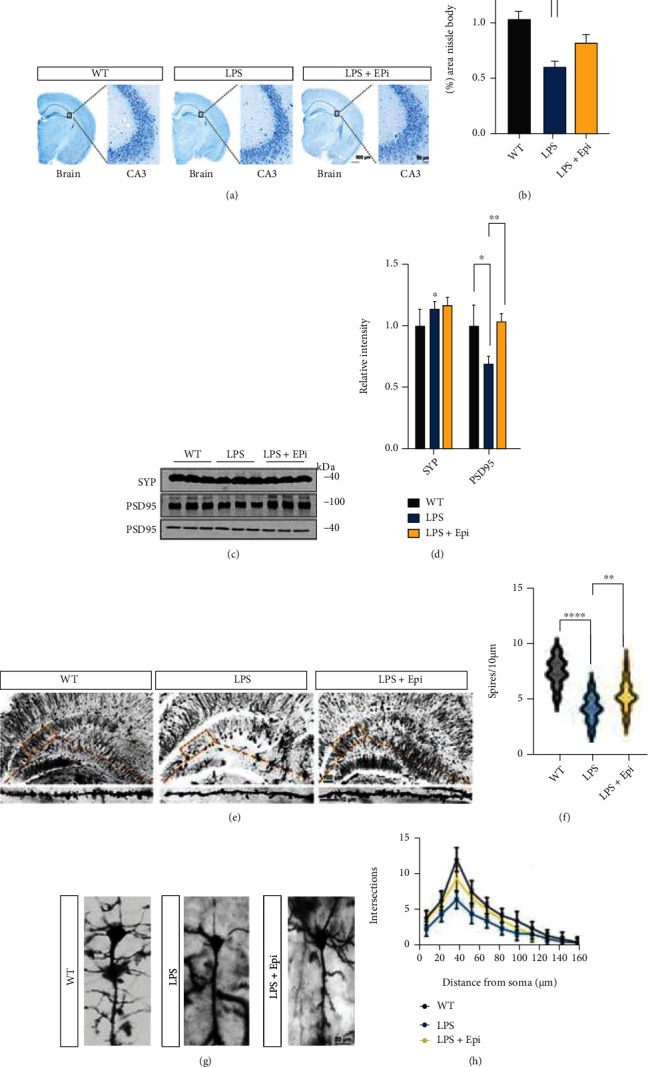
Epi treatment decreases neuronal and synaptic degeneration. (a) Representative Nissl staining images in the whole brain and in the CA3 of the hippocampus. (b) The ImageJ software was used to calculate the area of the Nissl body in the CA3 of the hippocampus. *n* = 3 mice/group. (c) Hippocampal synaptic protein levels were assessed by western blots. (d) The ratio of each synaptic protein to GAPDH was determined. *n* = 3 mice/group. (e) Representative images of Golgi-impregnated hippocampal neurons and magnified dendritic spines from mice in three groups. (f) Quantification of the spine density in (e). *n* = 21 from 3 mice/group. (g) Golgi staining images have shown the dendritic trees in mice. (h) The Sholl analysis was performed to evaluate the dendritic complexity of Epi or vehicle-treated mice. *n* = 21 from 3 mice/group. All data are shown as mean ± SEM. One-way ANOVA test followed by Tukey's post hoc test ^∗^*p* < 0.05, ^∗∗^*p* < 0.01, ^∗∗∗^*p* < 0.001, ^∗∗∗∗^*p* < 0.0001.

As Epi improves behavior damage, we tested the hypothesis that Epi treatment might reverse the loss of the hippocampal neuron and dendritic spine. Neuronal area measurements in the *CA3* subregion of the hippocampus after treatment demonstrated a significant decrease in the quantity of LPS compared to WT mice. In LPS mice, the application of Epi vs. vehicle resulted in a partial degree of protection from neuron loss (Figures 5(a) and 5(b)). Next, we explored the potential downstream effectors of Epi in mediating synapse in vivo. Western blot (WB) analysis was conducted to measure synaptosome fractions in hippocampal tissue samples using the synaptic markers synaptophysin (SYP) and PSD95. The results demonstrated a decrease in PSD95 in LPS vs. WT mice (Figures 5(c) and 5(d)). In LPS mice treated with Epi, drug exposure was associated with a significant increase in the PSD95 signal (Figures 5(c) and 5(d)). Furthermore, a similar result was found using counts of Golgi-stained spines in hippocampal neurons which demonstrated significant decreases in spine density in LPS vs. WT (Figures 5(e) and 5(f)). Cumulative frequency statistical analysis of spine density per dendrite demonstrated a notable increase in spine density in LPS+Epi mice compared to LPS alone. Moreover, we found that the Epi agent was able to restore the complexity of neuronal dendrites using Sholl analysis, which indicated more intersections of neurites in the Epi group than in the LPS group (Figures 5(g) and 5(h)). In summary, these data suggest that Epi rescued neuronal loss and synaptic disorders in LPS-treated mice.

### 3.6. Epi Inhibits Microglial Activation in Mice

**Figure 6:**
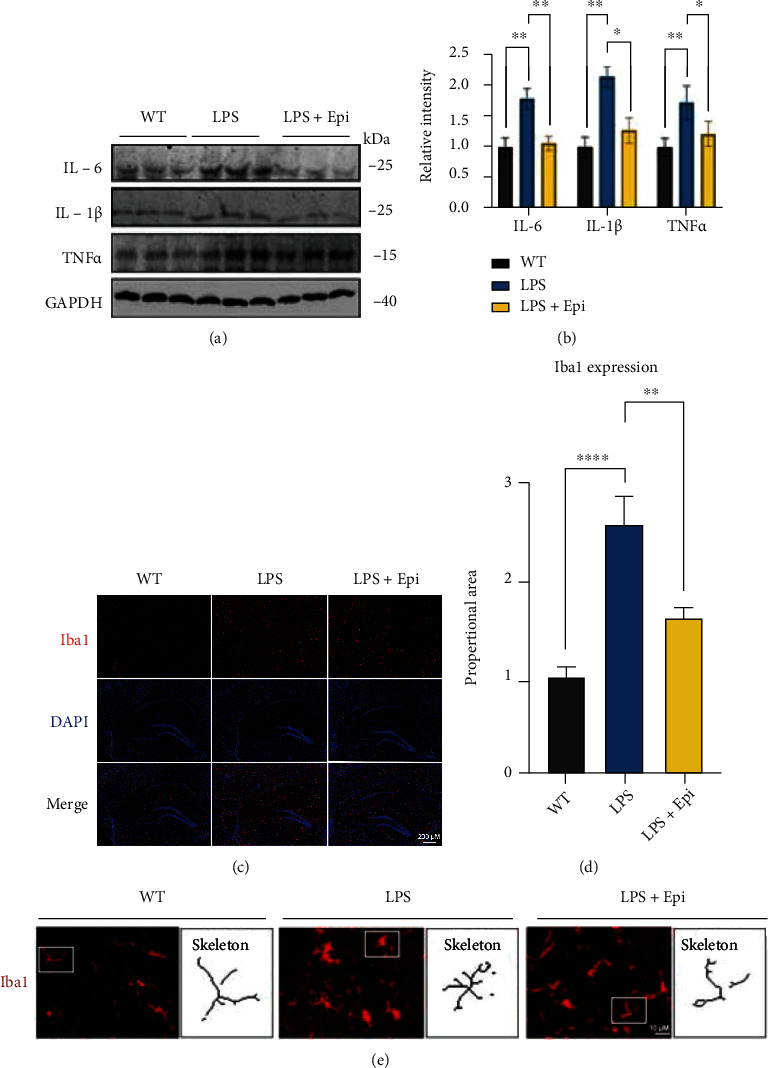
Epi intervention suppressed LPS-induced hippocampal microglial activation. (a) Expression of selected cytokine at the protein level in the hippocampus measured by western blotting. Quantification of data is shown in (b). (c, d) Images (×4) of Iba1 staining in the mouse hippocampus of three groups, with Iba1 area quantification. (e) Representative magnified profile of Iba1-labeled microglia morphology (×40) and process intersections by the Sholl profile analysis in the hippocampus. *n* = 3 mice/group. All data are shown as mean ± SEM. One-way ANOVA test followed by Tukey's post hoc test, ^∗^*p* < 0.05, ^∗∗^*p* < 0.01, ^∗∗∗∗^*p* < 0.0001.

Inflammatory cytokines are associated with activated microglia-mediated neuroinflammation. Thus, to investigate the potential regulatory effects of Epi on microglial activation, proinflammatory cytokine levels were measured by western blotting. LPS upregulated the levels of proinflammatory cytokines (Figures 6(a) and 6(b)). In contrast, Epi obviously downregulated the LPS-mediated increase in protein levels of IL-6, IL-1*β*, and TNF*α* in mice. To further confirm these findings, immunofluorescence was performed using anti-Iba1. In accordance with the above results, Epi significantly reduced LPS-induced Iba1 level compared to the LPS treatment (Figures 6(c)–6(e)). We found that microglia in wild-type mice were sparsely distributed in the hippocampus (Figures 6(c) and 6(d)). However, microglia in LPS-treated mice showed deepened and enlarged staining of cell bodies [42]. We also applied Sholl analysis quantification to summarize the representative microglial profile. The microglia in LPS mice were less ramified and amoeboid in shape, suggesting that they had become reactive. Moreover, the microglia in the Epi intervention group showed a more ramified and baseline quiescent state (Figure 6(e)). Our results showed that pretreatment with Epi inhibits LPS-induced inflammatory responses and suppresses microglial infiltration in mice.

### 3.7. Epi Rescues Mitochondrial Dysfunction in LPS-Treated Mice

**Figure 7:**
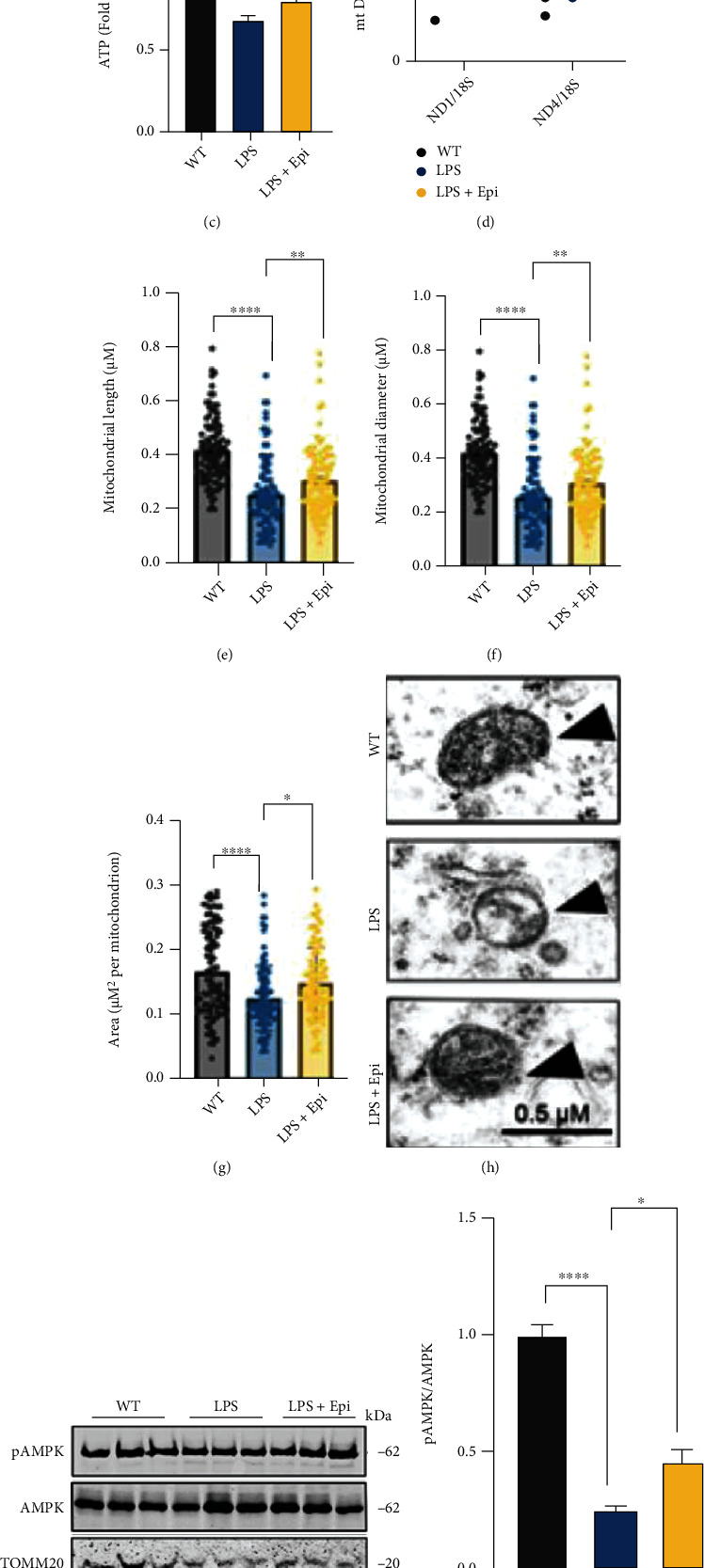
Epi might alleviate LPS-induced mitochondrial dysfunction via AMPK. (a–c) Levels of SOD, MDA, and ATP were detected in the hippocampus of the mouse model. (d) Epi increased the mt DNA copy number in LPS-induced mice by using qRT-PCR analysis. *n* = 3 mice/group. (e–g) Quantification of mitochondrial parameters from electron microscopy images in mice; *n* = 100 from 3 mice/group. (h) Representative set of electron microscopy images. (i) Relative levels of proteins implicated in the AMPK pathway and mitochondria marker TOMM20. Quantification of data is shown in (j, k). *n* = 3 mice/group. All data are shown as mean ± SEM. One-way ANOVA test followed by Tukey's post hoc test ^∗^*p* < 0.05, ^∗∗^*p* < 0.01, ^∗∗∗^*p* < 0.001, ^∗∗∗∗^*p* < 0.0001.

We examined whether Epi treatment affected the function of mitochondrial function. We found reduced expression of SOD and ATP and increased expression of MDA in the hippocampus of the LPS-treated mice. Interestingly, we also found increased expression of SOD and ATP and decreased expression of MDA in the Epi and LPS cotreated mice compared to the mice only treated with LPS (Figures 7(a)–7(c)), which indicates that Epi could improve mitochondrial function in the hippocampus of the LPS-treated mice. It is important to note that Epi treatment also promotes mitochondrial biogenesis (Figure 7(d)). Next, we examined the morphology of mitochondria in hippocampal neurons from three age- and sex-matched groups of mice. Hippocampal neurons in the LPS-treated mice displayed altered mitochondrial morphology characterized by smaller size and excessive mitochondrial damage compared to WT controls (Figures 7(e)–7(h)). Furthermore, Epi rescued mitochondrial impairment by maintaining mitochondrial morphology and reducing mitochondrial vacuolation (Figures 7(e)–7(h)). Mitochondrial dysfunction is defined as energy deficiency, which results in ATP depletion. Because AMPK is a key regulator of energy expenditure and mitochondrial homeostasis, we examined the protein levels of the AMPK signaling pathway. Using western blotting, we found that phosphorylation of AMPK (pAMPK) and translocase of outer mitochondrial membrane 20 (TOMM20) were decreased in the LPS group, suggesting that AMPK signaling was downregulated and mitochondria were impaired (Figures 7(i)–7(k)). Interestingly, there was an increase in AMPK activity after being treated with Epi (Figures 7(i) and 7(j)). We observed that the reactivation of AMPK restores the level of TOMM20 (Figures 7(i) and 7(k)). These results highlight that Epi may activate AMPK in response to mitochondrial impairment in the LPS-treated mice.

## 4. Discussion

Currently, there is no effective therapy for SAE partly because the underlying mechanisms involved are poorly defined. Manipulation of the AMPK signaling pathway has been considered to have potential as a treatment for SAE [43]. It has been reported that pAMPK expression is significantly reduced following LPS-induced brain injury [44]. Epi is a natural polyphenolic substance derived from dietary flavonoids and a potent neuroprotective candidate [8], which has been proven to be able to cross the blood-brain barrier and potentially act directly on neurons. Epi has no significant side effects identified in humans or mice [45]. Furthermore, Epi has been shown to increase blood flow by stimulating nitric oxide (NO) production, modifying the metabolic profile, and preventing antioxidative damage [46]. Thus, Epi is a promising neuroprotective intervention for many neurological diseases. In this study, we found that administration of Epi prior to LPS injury limits neuroinflammation. The microglial activation response often occurs as a result of LPS exposure. Microglia respond by promoting the secretion of proinflammatory cytokines (IL-1*β*, IL-6, TNF*α*, etc.), redox molecules (iNOS), and COX2 [47–49]. Previous studies have shown that Epi modulates the effect on IL-1*β*, IL6, IL-10, TNF*α*, iNOS, COX2, and NF-*κ*B [50, 51]. To explore the potential regulatory effects of Epi on the microglial activation response induced by LPS in this study, proinflammatory cytokine levels (IL-1*β*, IL-6, and TNF*α*) were measured using qRT-PCR and western blotting. We found that Epi suppressed the production of the above-mentioned proinflammatory cytokines in the LPS-treated models.

We also found that Epi upregulated pAMPK expression and that Epi lost its protective effect in AMPK-blocked cells, suggesting that the protective effect of Epi is mediated by the activation of AMPK signaling. We performed a pilot experiment in LPS-treated mice to determine the role of Epi in animals. We found that Epi also exerted a protective effect in LPS-treated mice models. In addition, we also provided in vivo evidence that administration of Epi reduced cognitive decline, reduced neuronal damage, and prevented neuronal dendritic spine loss. This result is consistent with a previous study which showed that Epi improved cognitive function and reduced neuronal damage in an Alzheimer's disease mouse model [52]. These improvements were associated with decreased neuroinflammation and increased mitochondrial quality. Moreover, the administration of Epi regulated the AMPK signaling pathway in vivo. Based on this evidence, we suggest that the administration of Epi-mediated activation of AMPK is a promising therapeutic approach against SAE.

The mechanisms of SAE include mitochondrial dysfunction, oxidative damage, neurotransmission disturbances, inflammation, and cell death [53]. Several studies in experimental models and patients have shown that mitochondrial dysfunction in various tissues is associated with the severity of sepsis [54, 55]. Mitochondrial dysfunction has been increasingly recognized as a mechanical link between neuroinflammation and cell damage during neuronal loss [56]. A previous study indicated that increased mitochondrial biogenesis under LPS-induced septic conditions could satisfy the high-energy demand and promote mitochondrial recovery [57]. In rats subjected to sepsis, mitochondrial protectors improve hippocampal mitochondrial function and reduce oxidative injury and neuroinflammation, thus leading to decreased long-term memory impairment [58].

There is a wealth of intervention studies linking the consumption of flavan-3-ols to the improvement of cognitive function [59]. In this context, Epi has been causally linked to these beneficial effects [45]. (–)-Epicatechin has protective effects against oxidative injury and mitochondrial damage. Epi reduced homocysteine-induced mitochondrial lipid peroxidation in the rat hippocampus [60]. Furthermore, Epi has been shown to have an important effect on cellular signaling upstream of mitochondrial regulation [61]. Moreover, Epi has also been shown to increase or modulate AMPK signaling in the previous study [62]. Epi pretreatment improved cell membrane stability and prevented the inactivation of the AMPK pathway [63, 64]. These studies highlight the importance of Epi in the protection of brain homeostasis. Our study indicates a protective effect of Epi in LPS-induced cell and mouse models. We demonstrated that LPS interferes with mitochondrial function, leading to loss of mitochondrial membrane potential, energy stress, activation of inflammation, and cell death. All these effects are greatly blunted by Epi treatment, as demonstrated by increased ATP and pAMPK levels as well as improved memory function. From our limited time-course studies, it seems that Epi may protect the mitochondria through the activation of AMPK signaling in SAE.

The current study has several limitations. First, several pathways may be activated by Epi, and AMPK-mediated mitochondrial control may be one of the regulatory pathways. Proteomic profiling of the hippocampus may provide further information to help solve this important issue. Second, we only tested one dose of Epi in LPS-treated mice and observed a significant ameliorative effect. This administration amount was estimated based on the recommended dosage of Epi for treating Alzheimer's disease in mice. Future studies should be conducted to test whether other drug concentrations of Epi would be more efficient in ameliorating cognitive impairment in SAE models. Third, in the present studies, we used young and male mice for pretreatment studies; to further determine the protective effects of Epi, we need to test the effects of Epi on age- and sex-matched mice after the LPS intervention for therapy studies. In addition, the effect of Epi on LPS-induced anti-inflammatory factors, such as IL-10, IL-4, and IL-13, needs to be further explored.

Overall, our study provides insight into the benefits of Epi in regulating mitochondrial quality control and neuroinflammation via the AMPK pathway to mitigate the effects of SAE in LPS-induced models. This suggests that Epi therapy may be a promising novel adjuvant modality to improve neurological prognosis in survivors of sepsis.

## Data Availability

The data used to support the findings of this study are included in the article.
